# A review of the genus *Scaponopselaphus* Scheerpeltz (Insecta: Coleoptera: Staphylinidae)

**DOI:** 10.3897/BDJ.3.e4735

**Published:** 2015-04-14

**Authors:** Stylianos Chatzimanolis

**Affiliations:** ‡ University of Tennessee at Chattanooga, Chattanooga, United States of America

**Keywords:** Xanthopygina, Staphylininae, Staphylinini, neotropics

## Abstract

**Background:**

The genus *Scaponopselaphus* Scheerpeltz was originally described to accommodate the species *Trigonopselaphus mutator* Sharp.

**New information:**

In this paper, I review *Scaponopselaphus* and describe a new species from Colombia as *Scaponopselaphus diaspartos* n. sp. Illustrations are provided for the identification of specimens and the presence of spatulate setae on first mesotarsomere is shown to be a unique characteristic of *Scaponopselaphus* within Xanthopygina.

## Introduction

Between 1870 and 1940, the genus *Trigonopselaphus* Gemminger and Harold was treated as a dumping ground for species with securiform labial palpomere 3 and metallic coloration. Eventually species were moved out of *Trigonopselaphus* and into other genera such as *Gastrisus* Sharp, *Nausicotus* Sharp, and *Torobus* Herman (see Herman 2001 for the catalogue and Chatzimanolis 2013 for the morphogroups in *Torobus*), but great uncertainty still exists regarding the taxonomic boundaries of these genera. Recently, I have started dealing with the taxa originally placed in *Trigonopselaphus*: I erected the genus *Terataki* Chatzimanolis (Chatzimanolis 2013) to deal with several taxa that had been moved to *Torobus* and I revised the genus *Trigonopselaphus* (submitted) to establish the limits in that genus.

In this paper, I review the genus *Scaponopselaphus* Scheerpeltz. *Scaponopselaphus* was described (Scheerpeltz 1972) based on a single species and single specimen, *Trigonopselaphus mutator* Sharp, 1876. Sharp himself contemplated whether or not he should place the species in *Trigonopselaphus* (sensu Sharp 1876; currently this would be *Torobus* Herman) or erect a new genus for it, given that he thought that the absence of postcoxal process distinguished it from other species in *Trigonopselaphus*. Sharp (1876, p. 145) decided to place the species in *Trigonopselaphus* since "[*Trigonopselaphus*] has already scarcely any definite meaning”. Here, I reaffirm the generic status of *Scaponopselaphus* by providing novel morphological characters and I also describe a new species from Colombia.

## Materials and methods

Photographs were taken using a Visionary Digital Passport System with a Canon EOS 40D camera and MP-E 65 lens. Images were automontaged using Helicon Focus 6.2.2. SEM photographs were taken using a Neoscope JEOL desktop SEM and processed using the Fluid Mask 3 software. All specimens were examined using an Olympus SZX10 stereomicroscope. Measurements were made using an ocular micrometer. Width: length ratio measurements were made on the widest and longest parts of the structure. The comparison between the length of the median lobe and the paramere excludes the bulbous basal part of the median lobe. Total body length is measured from the anterior margin of frons to the posterior margin of tergite VIII. Terminology and label data follow the procedure established by Ashe and Chatzimanolis 2003 and used in other Xanthopygina taxonomic works (e.g., Chatzimanolis 2004, Chatzimanolis 2008, Chatzimanolis 2012, Chatzimanolis 2013, Chatzimanolis 2014a, Chatzimanolis and Ashe 2009). The type locality of *Scaponopselaphus mutator* (Sharp) has been corrected as indicated in Asenjo et al. 2013.

Depositories:

- BMNH - The Natural History Museum, London, UK (R. Booth);
- FMNH - Field Museum of Natural History, Chicago, IL, USA (A. Newton, M. Thayer);
- SEMC - Snow Entomological Collection, University of Kansas, Lawrence, KS, USA (Z. Falin);
- UTCI - University of Tennessee at Chattanooga Insect Collection, Chattanooga, TN, USA (S. Chatzimanolis).

## Taxon treatments

### Scaponopselaphus

Scheerpeltz, 1972

Scaponopselaphus Scheerpeltz, 1972: 38
Scaponopselaphus Scaponopselaphus mutator (Sharp, 1876)Sharp 1876: 144. original designation by Scheerpeltz 1972

#### Description

Redescription: Habitus as in Fig. 1, body medium sized, 10.1-10.8 mm in total length. Color of head and pronotum metallic blue, green or purple blue; elytra light brown to brown; ventral surface of body light brown to brown. Mouthparts orange; antenna orange to brown; abdomen light brown to brown exceptVIII and posterior part of VII orange.

Head transverse (Figs 2, 4a), with medium-sized setose punctures and distinctive microsculpture (Fig. 4a) in microlines; epicranium shining, with large prominent macrosetae along border of head. Clypeus emarginate; anteclypeus expanded, well developed. Eyes large, prominent, occupying more than 3/4 of lateral margins of head. Ventral surface of head with transverse microsculpture; postoccipital suture and ventral basal ridge present; infraorbital ridge pronounced posteriorly; postmandiblular ridge present, prominent, extending from near mandible to posterior border of head; gular sutures separated throughout length with narrowest point between them near mid-length; nuchal depression prominent forming well defined neck; neck with microsculpture and few micropunctures.

Antenna (Fig. 4b). Antennomeres 1-3 with multiple rows of macrosetae; antennomeres 4-11 with few macrosetae but covered with microtrichia; antennomeres 1-3 longer than wide; antennomere 4 quadrate; antennomeres 5-10 subquadrate to transverse, just slightly asymmetrical, becoming wider towards antennomere 10; antennomere 11 longer than wide.

Mouthparts. Labrum (Fig. 4a) medially incised. Mandibles as in Fig. 3a, b; curved, moderately elongate, with short tooth medially; left and right mandibles nearly symmetrical; with lateral fold extending from condyle to tooth; prostheca setose. Maxilla as in Fig. 3d; galea and lacinia densely setose; maxillary palpi 4-segmented; P_1_ small, about 1/3 as long as P_2_; P_2_ curved, elongate, subequal in length to P_3_; P_2_-P_3_ with large setae apically; P_4 _elongate, slightly longer than P_3_. Labium as in Fig. 3c; mentum with one long and one shorter anterolateral setae at each end. Labial palpi 3-segmented; with transverse microsculpture; P_1_ longer than P_2_; P_2_ trapezoidal; both P_1_ and P_2_ with several long setae; P_3_ securiform; P_3_ apex wide and with 4-5 rows of sensory setae.

Pronotum subquadrate (Fig. 2); hypomeron expanded (Fig. 4c), with microsculpture; superior and inferior marginal lines of hypomeron separate throughout their lengths; superior line fully visible from above, extending around anterolateral margin of pronotum and contacting inferior line at neck fossa; no portion of dorsum of pronotum visible from below; without postcoxal process. Surface of pronotum shining, with scattered large setose punctures and microsculpture made of microlines (similar to but not as dense as on head); punctures on pronotum denser near anterolateral corners; margins of pronotum with several large setae. Mesoscutellum with dense polygon-shaped microsculpture and multiple rows of small punctures. Basisternum (Fig. 4c) with dense polygon-shaped microsculpture and weak carina; anterior marginal depression present; furcasternum with medial carina pointed vertically; furcasternum without microsculpture.

Elytra subequal to pronotum; with confluent or almost confluent punctures and large setae; with micropunctures but no other microsculpture; elytra appearing shining. Hind wings fully developed. Mesoventrite (Fig. 4d) with anterior margin forming “lip”; with dense polygon-shaped microsculpture and few punctures along edges; without median carina. Metaventrite (Fig. 4d) with dense uniform medium-sized punctures; metaventral process small, rounded, triangular.

Legs. Tarsal segmentation 5-5-5; pro- and mesofemur in both sexes with ctenidium ventrally and proximally; meso- and metatibia with multiple rows of spurs; protibia without multiple rows of spurs but with single row of spurs apically. Protarsus (Fig. 5d​) enlarged in both sexes, with spatulate setae ventrally; mesotarsus (Fig. 5e) not enlarged except tarsomere 1 in males twice as wide as other mesotarsomeres and with spatulate setae ventrally; metatarsus not enlarged. Empodium with two small setae.

Abdomen with paired protergal glands present; expanding from segment III to segment V (widest) and then becoming narrower towards segment VIII. Abdominal tergites III-V (Fig. 5a) with tergal basal carina and curved (arch-like) carina. Tergites and sternites with distinctive microsculpture (Fig. 5a, b) on anterolateral corners, sometimes expanded medially. Males with secondary sexual structures of sternites VII-IX (Fig. 5c): sternite VII with round porose structure anteriorly and U-shaped emargination posteriorly; sternite VIII with deep U-shaped emargination posteriorly; sternite IX with V-shaped emargination.

Male and Female Genitalia. Aedeagus typical of Xanthopygina (Figs 6, 7); with long median lobe and paramere divided into two lobes. Paramere with peg setae and short apical setae. Spermatheca not sclerotized.

#### Diagnosis

*Scaponopselaphus* can be distinguished from all other genera in Xanthopygina by the combination of the following characters: (1) Head with distinctive microsculpture (Fig. 4a); (2) labial palpomere 3 (P_3_) securiform (Fig. 3c); (3) pronotum with broad and convex lateral margins (Fig. 2); (4) mesotarsomere 1 in males with spatulate setae [unknown in other Xanthopygina] (Fig. 5e); (5) tergites III-V with curved (arch-like) carina (Fig. 5a); and (6) sternite VII in males with small porose structure (Fig. 5c). Male specimens in *Scaponopselaphus* can always be easily identified by the spatulate state on mesotarsomere 1, but some species in *Phanolinopsis* Scheerpeltz, *Styngetus* Sharp, *Xenopygus* Bernhauer may look superficially like *Scaponopselaphus*. However, these taxa do not have securiform labial P_3_ and their pronotum is not convex. Perhaps the most confusing scenario can be if someone has unsorted female specimens of *Scaponopselaphus*, *Torobus* and *Zackfalinus* Chatzimanolis; all these taxa have securiform labial P_3_ and somewhat similar head. However, *Scaponopselaphus* can be distinguished from these two genera based on the microsculpture of the head and the shape of the pronotum.

#### Distribution

**Figure 8.**
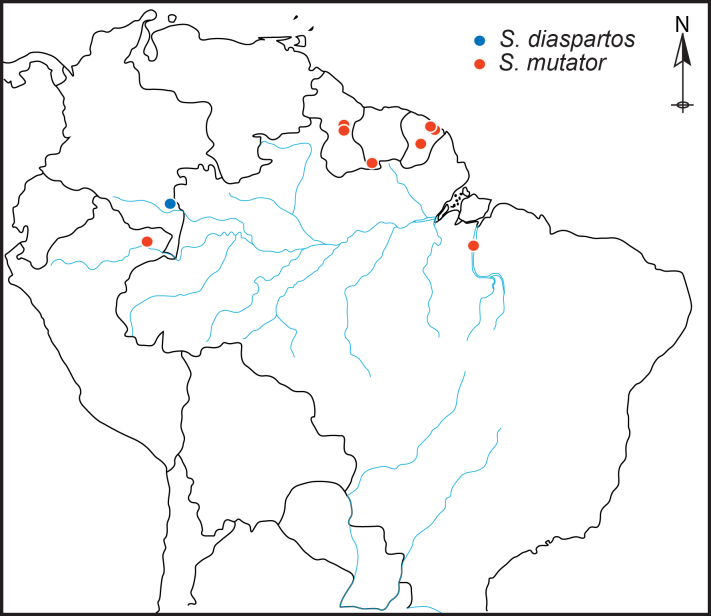
Distribution map of *S. diaspartos* Chatzimanolis and *S. mutator* (Sharp).

Known from the state of Pará in Brazil, the department of Vaupés in Colombia, the province of Sucumbios in Ecuador, French Guiana, Guyana, the departments of Loreto and Madre de Dios in Peru and from Suriname (Fig. 8).

#### Ecology

Specimens of *Scaponopselaphus* have been collected from wet tropical lowlands, however, further details on their habitat are unkown since almost all taxa have been collected with malaise or flight intercept traps. It is possible that the genus prefers forested habitats near rivers based on recent collecting events.

### Scaponopselaphus diaspartos

Chatzimanolis, 2015 sp. n.

urn:lsid:zoobank.org:act:85B7B681-1090-46EB-AF00-B56F5E842973

#### Materials

- **Type status:** Holotype. **Occurrence:** catalogNumber: SM0645233; recordedBy: L. Benavides; individualCount: 1; sex: male; lifeStage: adult; **Taxon:** scientificName: Scaponopselaphus diaspartos; **Location:** country: Colombia; stateProvince: Vaupés; locality: Mosiro-Itajura (Caparú) Igapo; verbatimLocality: Colombia: Vaupés R. N., Mosiro-Itajura (Caparú) Igapo, 1°4'S 69°31'W, 60m, Malaise, 2-22.ix.2002, l. Benavides Leg. M.3393; verbatimElevation: 60 m; verbatimCoordinates: 1°4'S 69°31'W; decimalLatitude: -1.0666667; decimalLongitude: -69.5166667; georeferenceProtocol: label; **Identification:** identifiedBy: Stylianos Chatzimanolis; dateIdentified: 2015; **Event:** samplingProtocol: Malaise; eventDate: 2002-09-7/22; fieldNumber: M.3393; **Record Level:** institutionID: SEMC; basisOfRecord: PreservedSpecimen
- **Type status:** Paratype. **Occurrence:** catalogNumber: SM0628644; recordedBy: L. Benavides; individualCount: 1; sex: male; lifeStage: adult; **Taxon:** scientificName: Scaponopselaphus diaspartos; **Location:** country: Colombia; stateProvince: Vaupés; locality: Mosiro-Itajura (Caparú) Igapo; verbatimElevation: 60 m; verbatimCoordinates: 1°4'S 69°31'W; decimalLatitude: -1.0666667; decimalLongitude: -69.5166667; georeferenceProtocol: label; **Identification:** identifiedBy: Stylianos Chatzimanolis; dateIdentified: 2015; **Event:** samplingProtocol: Malaise; eventDate: 2002-11-2/22; fieldNumber: M.3397; **Record Level:** institutionID: SEMC; basisOfRecord: PreservedSpecimen
- **Type status:** Paratype. **Occurrence:** catalogNumber: SM0628643; recordedBy: L. Benavides; individualCount: 1; sex: female; lifeStage: adult; **Taxon:** scientificName: Scaponopselaphus diaspartos; **Location:** country: Colombia; stateProvince: Vaupés; locality: Mosiro-Itajura (Caparú) Igapo; verbatimElevation: 60 m; verbatimCoordinates: 1°4'S 69°31'W; decimalLatitude: -1.0666667; decimalLongitude: -69.5166667; georeferenceProtocol: label; **Identification:** identifiedBy: Stylianos Chatzimanolis; dateIdentified: 2015; **Event:** samplingProtocol: Malaise; eventDate: 2002-11-2/22; fieldNumber: M.3397; **Record Level:** institutionID: SEMC; basisOfRecord: PreservedSpecimen
- **Type status:** Paratype. **Occurrence:** catalogNumber: UTCI000004901; recordedBy: L. Benavides; individualCount: 1; sex: female; lifeStage: adult; otherCatalogNumbers: 8c1dfaaa-dc0c-4581-a3cb-3cce16bdd17e; **Taxon:** scientificName: Scaponopselaphus diaspartos; **Location:** country: Colombia; stateProvince: Vaupés; locality: Estación Biológica Mosiro-Itajura (Caparú) Antigua Cabaña; verbatimElevation: 60 m; verbatimCoordinates: 1°4'S 69°31'W; decimalLatitude: -1.0666667; decimalLongitude: -69.5166667; georeferenceProtocol: label; **Identification:** identifiedBy: Stylianos Chatzimanolis; dateIdentified: 2015; **Event:** samplingProtocol: Malaise; eventDate: 2003-02-01/09; fieldNumber: M.3612; **Record Level:** institutionID: UTC; collectionID: UTCI; basisOfRecord: PreservedSpecimen; source: http://symbiota4.acis.ufl.edu/scan/portal/collections/individual/index.php?occid=13641794&clid=0

#### Description

Habitus as in Fig. 1a. Body length 10.3-10.8 mm. Coloration of head and pronotum dark metallic purple-blue (Fig. 2a); antennae and mouthparts orange; elytra and abdomen shiny brown, except intersegmental membranes yellow, sternite VIII and posterior 1/4 of sternite VIII orange; legs and pronotal hypomeron orange-brown.

Head transverse, width: length ratio = 1.47; surface of epicranium flat; with medium-sized umbilicate punctures throughout surface except medially, distance between punctures varies but typically equals diameter of puncture. Eyes large, length of eyes / length of head = 0.58, distance between eyes as wide as 1.44 times length of eye.

Pronotum subquadrate, width: length ratio = 1.13; with scattered large umbilicate punctures, distance between punctures varies but typically equals 0.5-1 times diameter of puncture. Mesoscutellum with medium-sized punctures, punctures not confluent. Elytra with large, almost confluent punctures; each row with approximately 11 punctures (measured at middle of elytron). Abdominal tergites III-V with strongly delineated curved (arch-like) carina.

Secondary sexual structures. Males with posterior border of sternite VII with deep U-shaped emargination; sternite VIII with deep, broad U-shaped emargination medially; sternite IX with deep V-shaped emargination medially. Females with no obvious secondary sexual structures. Aedeagus as in Fig. 6; paramere divided to near base into two lobes; lobes narrower and subequal in length to median lobe; in dorsal view each lobe converging to rounded apex; in lateral view paramere slightly convex; with peg setae (sensory spinules) as shown in Fig. 6c, scattered throughout length of two lobes. Median lobe in dorsal view converging to narrow pointed apex; with single narrow dorsal tooth; in lateral view becoming much narrower near apex.

#### Diagnosis

*Scaponopselaphus diaspartos* can be distinguished from *S. mutator* based on the following characters: epicranium flatter and distance between eyes longer in *S. diaspartos* than in *S. mutator*; pronotum punctation more dense in *S. diaspartos* than in *S. mutator* (Fig. 2); and elytra punctation more sparse in *S. diaspartos* than in *S. mutator* (Fig. 1). Additionally, the following characters can be used to distinguish between males of the two species: in *S. diaspartos* posterior border of sternite VII with deeper median emargination than in *S. mutator*; in *S. diaspartos* peg setae are more scattered in the paramere (Fig. 6c) than the peg setae of *S. mutator* (Fig. 7c); and the median lobe of in *S. diaspartos* is as long as the paramere (Fig. 6a) while the median lobe in *S. mutator* is longer than the paramere (Fig. 7a).

#### Etymology

The specific epithet is derived from the modern Greek word διάσπαρτος (scattered) and refers to the distribution of the peg setae on the parameres. The epithet is a noun in apposition.

#### Distribution

Known from Vaupés, Colombia.

### Scaponopselaphus mutator

(Sharp, 1876)

KF178800, KF178770, KF178755, KF178741, KF178725

Trigonopselaphus mutator Sharp, 1876: 144

#### Materials

- **Type status:** Holotype. **Occurrence:** individualCount: 1; sex: female; lifeStage: adult; **Taxon:** scientificName: Scaponopselaphus mutator; **Location:** country: Peru; stateProvince: Loreto; locality: Pebas; verbatimLocality: South America, Brazil/Type/Pebas/Sharp Coll 1905-313/*Trigonopselaphus mutator*, Type, amazons, D.S./Holotype *Trigonopselaphus mutator* Sharp, 1876 det. R.G. Booth 2011; locationRemarks: Sharp listed the specimen as being from Brazil, however, as pointed out by Asenjo et al. 2013, Pebas is in Peru, not Brazil; decimalLatitude: -3.31666667; decimalLongitude: -71.85; georeferenceSources: Asenjo et al. 2013; **Identification:** identifiedBy: Stylianos Chatzimanolis; dateIdentified: 2015; **Record Level:** institutionCode: BMNH; basisOfRecord: PreservedSpecimen
- **Type status:** Other material. **Occurrence:** recordedBy: M. Alvarenga; individualCount: 1; sex: female; lifeStage: adult; **Taxon:** scientificName: Scaponopselaphus mutator; **Location:** country: Brazil; stateProvince: Pará; locality: Tucurui; decimalLatitude: -3.768; decimalLongitude: -49.673; coordinateUncertaintyInMeters: 3036; georeferenceProtocol: GEOLocate; **Identification:** identifiedBy: Stylianos Chatzimanolis; dateIdentified: 2015; **Event:** verbatimEventDate: i.1979; **Record Level:** institutionCode: FMNH; basisOfRecord: PreservedSpecimen
- **Type status:** Other material. **Occurrence:** catalogNumber: SM0099817; recordedBy: J. Ashe, R. Brooks; individualCount: 1; sex: male; lifeStage: adult; **Taxon:** scientificName: Scaponopselaphus mutator; **Location:** country: French Guiana; locality: Saül, Mt. Galbao summit; verbatimElevation: 740 m; verbatimCoordinates: 3°37'18''N 53°16'42''W; decimalLatitude: 3.6216667; decimalLongitude: -53.2783333; georeferenceProtocol: label; **Identification:** identifiedBy: Stylianos Chatzimanolis; dateIdentified: 2015; **Event:** samplingProtocol: flight intercept trap; eventDate: 1997-06-05/07; fieldNumber: FG1AB97 154; **Record Level:** institutionCode: SEMC; basisOfRecord: PreservedSpecimen
- **Type status:** Other material. **Occurrence:** catalogNumber: SM0094899; recordedBy: J. Ashe, R. Brooks; individualCount: 1; sex: male; lifeStage: adult; **Taxon:** scientificName: Scaponopselaphus mutator; **Location:** country: French Guiana; locality: 8.4km SSE Roura; verbatimElevation: 200 m; verbatimCoordinates: 4°40'41''N 52°13'26''W; decimalLatitude: 4.6780556; decimalLongitude: -52.2238889; georeferenceProtocol: label; **Identification:** identifiedBy: Stylianos Chatzimanolis; dateIdentified: 2015; **Event:** samplingProtocol: flight intercept trap; eventDate: 1997-05-22/23; fieldNumber: FG1AB97 011; **Record Level:** institutionCode: SEMC; basisOfRecord: PreservedSpecimen
- **Type status:** Other material. **Occurrence:** catalogNumber: SM0100262; recordedBy: J. Ashe, R. Brooks; individualCount: 1; sex: male; lifeStage: adult; **Taxon:** scientificName: Scaponopselaphus mutator; **Location:** country: French Guiana; locality: Cayenne, 33.5km S and 8.4km NW Hwy N2 on HWY D5; verbatimElevation: 30 m; verbatimCoordinates: 4°48'18''N 52°28'41''W; decimalLatitude: 4.805; decimalLongitude: -52.4780556; georeferenceProtocol: label; **Identification:** identifiedBy: Stylianos Chatzimanolis; dateIdentified: 2015; **Event:** samplingProtocol: flight intercept trap; verbatimEventDate: 29.05-09.06.1997; fieldNumber: FG1AB97 171; **Record Level:** institutionCode: SEMC; basisOfRecord: PreservedSpecimen
- **Type status:** Other material. **Occurrence:** catalogNumber: SM0098930; recordedBy: J. Ashe, R. Brooks; individualCount: 1; sex: female; lifeStage: adult; **Taxon:** scientificName: Scaponopselaphus mutator; **Location:** country: French Guiana; locality: Saül, 7km N, 1km NW Les Eaux Claires, along Rue de Belizon trail; verbatimElevation: 280 m; verbatimCoordinates: 3°39'46''N 53°13'19''W; decimalLatitude: 3.6627778; decimalLongitude: -53.2219444; georeferenceProtocol: label; **Identification:** identifiedBy: Stylianos Chatzimanolis; dateIdentified: 2015; **Event:** samplingProtocol: flight intercept trap; eventDate: 1997-06-04/08; fieldNumber: FG1AB97 167; **Record Level:** institutionCode: SEMC; basisOfRecord: PreservedSpecimen
- **Type status:** Other material. **Occurrence:** catalogNumber: SM0253154; recordedBy: R. Brooks, Z. Falin; individualCount: 1; sex: male; lifeStage: adult; **Taxon:** scientificName: Scaponopselaphus mutator; **Location:** country: Guyana; stateProvince: Region 8; locality: Iwokrama Forest, 1km W Kurupukari, Iwokrama Field Station; verbatimElevation: 60 m; verbatimCoordinates: 4°40'19''N 58°41'4''W; decimalLatitude: 4.67184; decimalLongitude: -58.684; georeferenceProtocol: label; **Identification:** identifiedBy: Stylianos Chatzimanolis; dateIdentified: 2015; **Event:** samplingProtocol: flight intercept trap; eventDate: 2001-05-26/29; fieldNumber: GUY1BF01 064; **Record Level:** institutionCode: SEMC; basisOfRecord: PreservedSpecimen
- **Type status:** Other material. **Occurrence:** catalogNumber: SM0253407; recordedBy: R. Brooks, Z. Falin; individualCount: 1; sex: male; lifeStage: adult; **Taxon:** scientificName: Scaponopselaphus mutator; **Location:** country: Guyana; stateProvince: Region 8; locality: Iwokrama Forest, Kabocalli Field Station; verbatimElevation: 60 m; verbatimCoordinates: 4°17'4''N 58°30'35''W; decimalLatitude: 4.2844444; decimalLongitude: -58.509722; georeferenceProtocol: label; **Identification:** identifiedBy: Stylianos Chatzimanolis; dateIdentified: 2015; **Event:** samplingProtocol: flight intercept trap; eventDate: 2001-06-03/05; fieldNumber: GUY1BF01 146; **Record Level:** institutionCode: SEMC; basisOfRecord: PreservedSpecimen
- **Type status:** Other material. **Occurrence:** catalogNumber: SM0253421; recordedBy: R. Brooks, Z. Falin; individualCount: 1; sex: male; lifeStage: adult; **Taxon:** scientificName: Scaponopselaphus mutator; **Location:** country: Guyana; stateProvince: Region 8; locality: Iwokrama Forest, Kabocalli Field Station; verbatimElevation: 60 m; verbatimCoordinates: 4°17'4''N 58°30'35''W; decimalLatitude: 4.2844444; decimalLongitude: -58.509722; georeferenceProtocol: label; **Identification:** identifiedBy: Stylianos Chatzimanolis; dateIdentified: 2015; **Event:** samplingProtocol: flight intercept trap; eventDate: 2001-06-03/05; fieldNumber: GUY1BF01 146; **Record Level:** institutionCode: SEMC; basisOfRecord: PreservedSpecimen
- **Type status:** Other material. **Occurrence:** catalogNumber: SM0253433; recordedBy: R. Brooks, Z. Falin; individualCount: 1; sex: female; lifeStage: adult; **Taxon:** scientificName: Scaponopselaphus mutator; **Location:** country: Guyana; stateProvince: Region 8; locality: Iwokrama Forest, Kabocalli Field Station; verbatimElevation: 60 m; verbatimCoordinates: 4°17'4''N 58°30'35''W; decimalLatitude: 4.2844444; decimalLongitude: -58.509722; georeferenceProtocol: label; **Identification:** identifiedBy: Stylianos Chatzimanolis; dateIdentified: 2015; **Event:** samplingProtocol: flight intercept trap; eventDate: 2001-06-03/05; fieldNumber: GUY1BF01 146; **Record Level:** institutionCode: SEMC; basisOfRecord: PreservedSpecimen
- **Type status:** Other material. **Occurrence:** catalogNumber: UTCI000004900; recordedBy: Larsen, Short; individualCount: 1; sex: male; lifeStage: adult; otherCatalogNumbers: 3a0568e7-eb54-45de-b52c-a12944f03a7f, SC230; associatedSequences: KF178800, KF178770, KF178755, KF178741, KF178725; **Taxon:** scientificName: Scaponopselaphus mutator; **Location:** country: Suriname; stateProvince: Sipaliwini; locality: Camp 1: on Kutari River; verbatimElevation: 228 m; decimalLatitude: 2.17535; decimalLongitude: -56.7874; georeferenceProtocol: label; **Identification:** identifiedBy: Stylianos Chatzimanolis; dateIdentified: 2015; **Event:** samplingProtocol: flight intercept trap; eventDate: 2010-08-19/24; fieldNumber: SR10-0819-TN1; eventRemarks: 2010 CI-RAP Survey; **Record Level:** institutionCode: UTC; collectionCode: UTCI; basisOfRecord: PreservedSpecimen; source: http://symbiota4.acis.ufl.edu/scan/portal/collections/individual/index.php?occid=13640919&clid=0
- **Type status:** Other material. **Occurrence:** catalogNumber: SM0349335; recordedBy: Larsen, Short; individualCount: 1; sex: female; lifeStage: adult; otherCatalogNumbers: SC231; **Taxon:** scientificName: Scaponopselaphus mutator; **Location:** country: Suriname; stateProvince: Sipaliwini; locality: Camp 2: on Sipaliwini River; verbatimElevation: 210 m; decimalLatitude: 2.182883; decimalLongitude: -56.78725; georeferenceProtocol: label; **Identification:** identifiedBy: Stylianos Chatzimanolis; dateIdentified: 2015; **Event:** samplingProtocol: flight intercept trap; verbatimEventDate: 27.viii.-1.ix.2010; fieldNumber: SR10-0827-TN2; eventRemarks: 2010 CI-RAP Survey; **Record Level:** institutionCode: SEMC; basisOfRecord: PreservedSpecimen
- **Type status:** Other material. **Occurrence:** catalogNumber: SM0349336; recordedBy: Larsen, Short; individualCount: 1; sex: female; lifeStage: adult; **Taxon:** scientificName: Scaponopselaphus mutator; **Location:** country: Suriname; stateProvince: Sipaliwini; locality: Camp 1: on Kutari River; verbatimElevation: 228 m; decimalLatitude: 2.17535; decimalLongitude: -56.7874; georeferenceProtocol: label; **Identification:** identifiedBy: Stylianos Chatzimanolis; dateIdentified: 2015; **Event:** samplingProtocol: flight intercept trap; eventDate: 2010-08-19/24; fieldNumber: SR10-0819-TN1; eventRemarks: 2010 CI-RAP Survey; **Record Level:** institutionCode: SEMC; basisOfRecord: PreservedSpecimen

#### Description

Habitus as in Fig. 1b. Body length 10.1-10.5 mm. Coloration of head and pronotum metallic blue, green or blue-green (Fig. 2b); antennae and mouthparts orange, except antennomeres 4-11 covered with darker brown setae; elytra and abdomen light brown to brown, except intersegmental membranes yellow, sternite VIII and posterior 1/3 of sternite VIII orange; legs and pronotal hypomeron orange to brown.

Head transverse, width: length ratio = 1.38; surface of epicranium flat to slightly convex; with medium-sized umbilicate punctures throughout the surface except medially, distance between punctures varies but typically equals 1-2 times of puncture. Eyes large, length of eyes / length of head = 0.68, distance between eyes as wide as 1.28 times length of eye.

Pronotum subquadrate, width: length ratio = 1.10; with scattered large umbilicate punctures, distance between punctures varies but typically equals diameter of puncture. Mesoscutellum with confluent medium-sized punctures. Elytra with large, confluent punctures; each row with approximately 15 punctures (measured at middle of elytron). Abdominal tergites III-V with weakly delineated curved (arch-like) carina.

Secondary sexual structures (Fig. 5c). Males with posterior border of sternite VII with shallow U-shaped emargination; sternite VIII with deep [but not as deep as in *S. diaspartos*], broad U-shaped emargination medially; sternite IX with shallow V-shaped emargination medially. Females with no obvious secondary sexual structures. Aedeagus as in Fig. 7; parameres divided to base into two lobes; lobes narrower and shorter than median lobe; in dorsal view each lobe converging to rounded apex; in lateral view paramere slightly convex; with peg setae (sensory spinules) as shown in Fig. 7c, concentrated near apex of lobes. Median lobe in dorsal view converging to broad pointed apex; with wide bicuspid dorsal tooth; in lateral view becoming narrower near apex.

#### Diagnosis

See the Diagnosis of *S. diaspartos*.

#### Distribution

Known from the state of Pará in Brazil, French Guiana, Guyana, the department of Loreto in Peru and from Suriname

### Scaponopselaphus spp.

#### Materials

- **Type status:** Other material. **Occurrence:** catalogNumber: SM0210544; recordedBy: R. Brooks; individualCount: 1; sex: female; lifeStage: adult; **Taxon:** scientificName: Scaponopselaphus sp.; **Location:** country: Peru; stateProvince: Madre de Dios; locality: Pakitza Biological Station, Castanal Trail, Reserved zone, Manu National Park; verbatimElevation: 317 m; verbatimCoordinates: 11°56'41''S 71°17'0''W; decimalLatitude: -11.9447222; decimalLongitude: -71.2833333; georeferenceProtocol: label; **Identification:** identifiedBy: Stylianos Chatzimanolis; dateIdentified: 2015; **Event:** samplingProtocol: flight intercept trap; eventDate: 2000-10-15/16; fieldNumber: PERU1B00 013; **Record Level:** institutionCode: SEMC; basisOfRecord: PreservedSpecimen
- **Type status:** Other material. **Occurrence:** catalogNumber: SM0153382; recordedBy: R. Brooks; individualCount: 1; sex: female; lifeStage: adult; **Taxon:** scientificName: Scaponopselaphus sp.; **Location:** country: Ecuador; stateProvince: Sucumbios; locality: Sacha Lodge; verbatimElevation: 270 m; verbatimCoordinates: 0°28'14''S 76°27'35''W; decimalLatitude: -0.4705556; decimalLongitude: -76.4597222; georeferenceProtocol: label; **Identification:** identifiedBy: Stylianos Chatzimanolis; dateIdentified: 2015; **Event:** samplingProtocol: flight intercept trap; eventDate: 1999-03-21/24; fieldNumber: ECU1B99 047; **Record Level:** institutionCode: SEMC; basisOfRecord: PreservedSpecimen

#### Notes

These two specimens look rather similar to *S. mutator*, however, I am unable to place them in that species without male specimens from the same locality.

## Discussion

In the recently completed molecular phylogeny of Xanthopygina, *Scaponopselaphus* was shown to be the sister group of *Elmas* Blackwelder (Chatzimanolis 2014b​). *Elmas* is a rather distinctive genus of xanthopygine rove beetles with several unique morphological features (see Ashe and Chatzimanolis 2003, Ashe and Chatzimanolis 2006 for details) and at first glance, *Elmas* and *Scaponopselaphus* do not share many morphological characteristics. While a morphological phylogeny of Xanthopygina is still in preparation and a list of synapomorphies for the two genera is not currently available, there are certain common features worth mentioning here. First of all, the overall bauplan of the head for both genera is similar. Both genera have securiform labial palpus 3, which appears rather similar, and it is unlike the securiform palpus of *Zackfalinus* or *Dysanellus* Bernhauer (see Chatzimanolis 2012 for details on the morphology). Also, the secondary sexual structures on sternites VII-IX have the same kind of medial emarginations, although it is worth pointing out here that *Elmas* does not have a porose structure on sternite VII as in *Scaponopselaphus*.

*Scaponopselaphus* does not appear to be very common in collections around the world. During the last 15 years, I was able to locate the genus only in the four museum collections listed in the Materials and Methods sections as depositories, even though I have visited most major museums in North America and Europe. However, I doubt that the genus is rare in the field and it is more likely that we have not sampled adequately at the correct habitat. Based on recent collecting events, it appears that *Scaponopselaphus* is easily collected with flight intercept traps in localities near rivers and it is quite likely that many more new species are awaiting discovery in South America.

## Supplementary Material

XML Treatment for Scaponopselaphus

XML Treatment for Scaponopselaphus diaspartos

XML Treatment for Scaponopselaphus mutator

XML Treatment for Scaponopselaphus spp.

## Figures and Tables

**Figure 1a.**
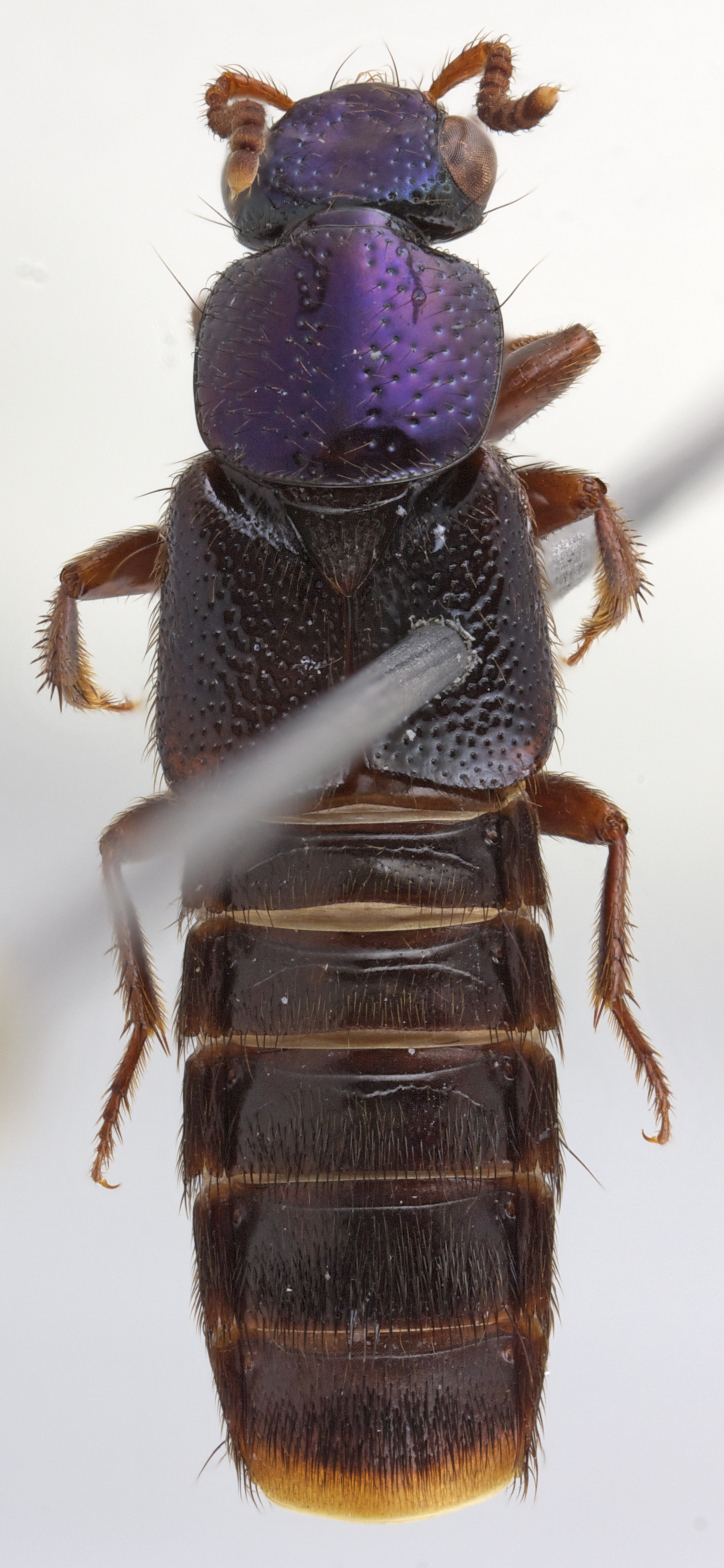
The holotype of *Scaponopselaphus diaspartos* Chatzimanolis

**Figure 1b.**
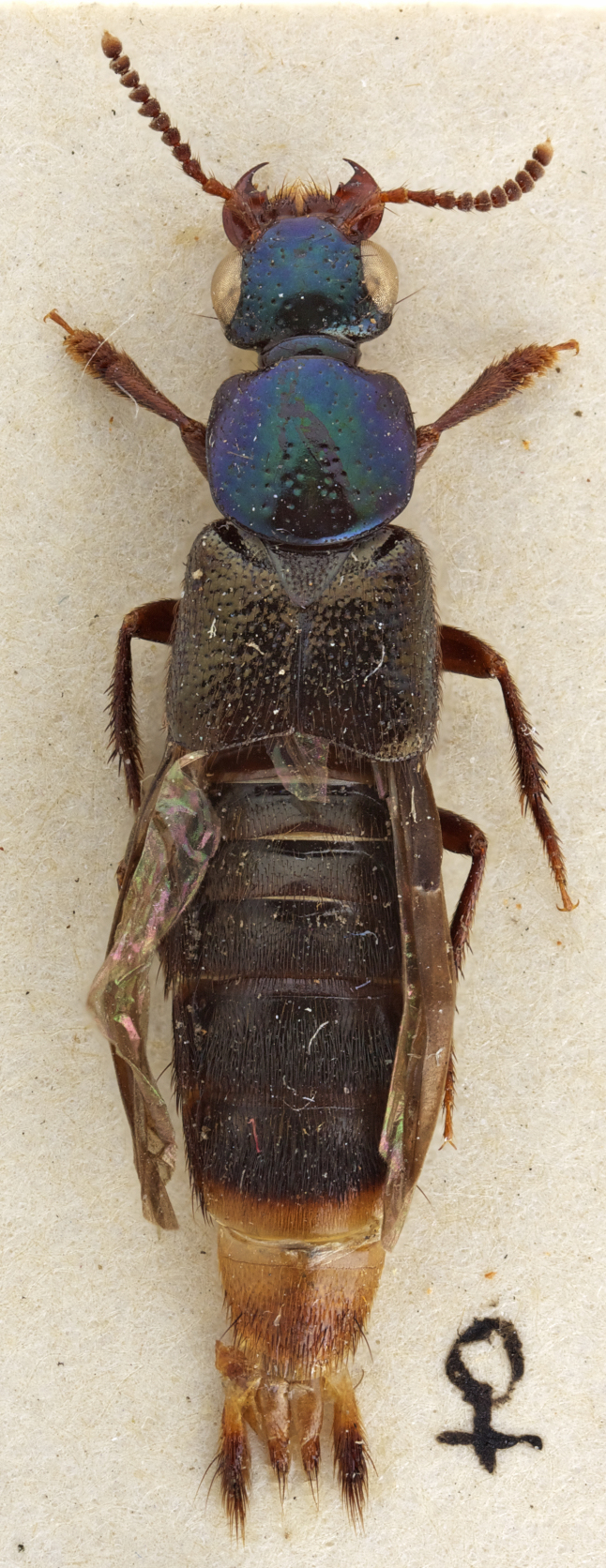
The holotype of *Scaponopselaphus mutator* (Sharp)

**Figure 2a.**
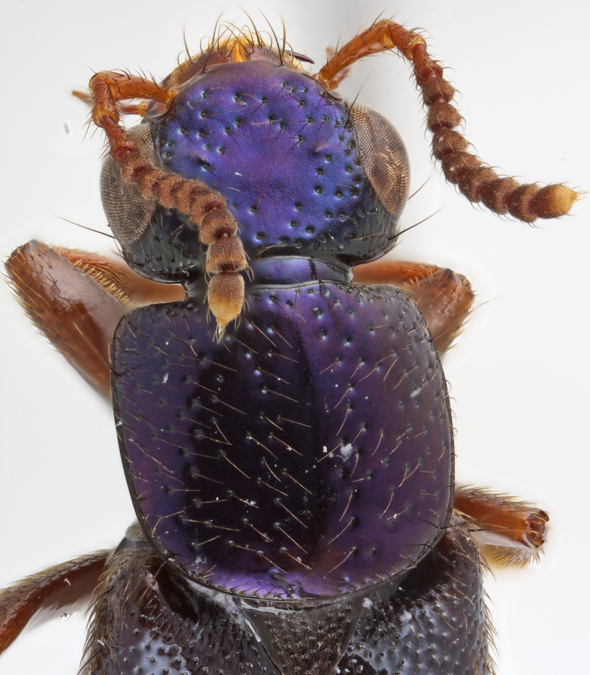
*S. diaspartos* Chatzimanolis

**Figure 2b.**
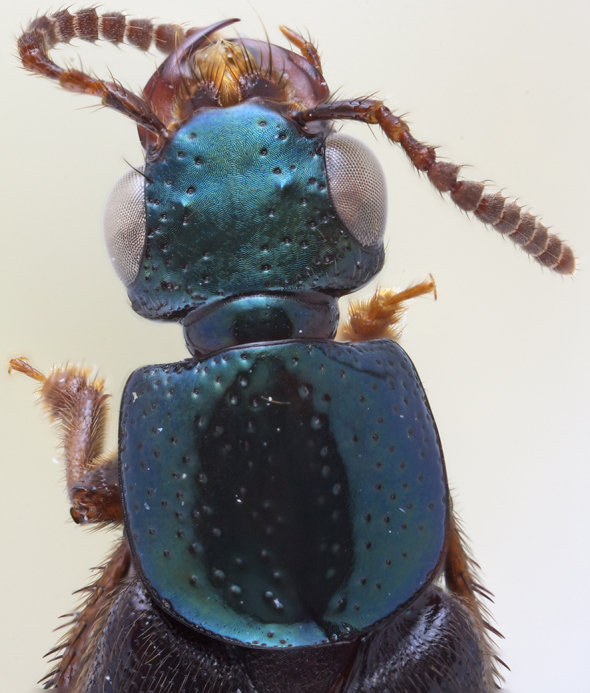
*S. mutator* (Sharp)

**Figure 3a.**
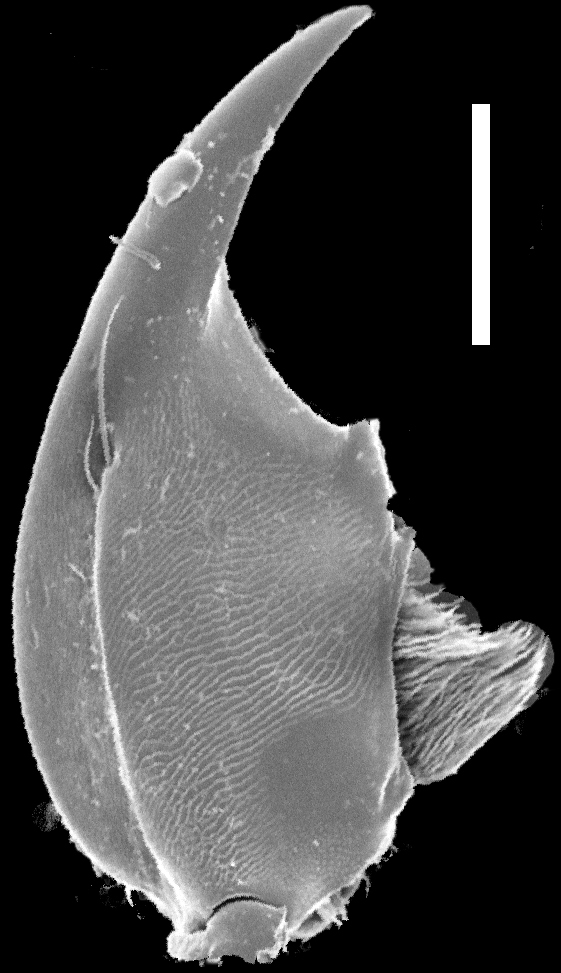
Dorsal view of left mandible. Scale bar = 0.25 mm.

**Figure 3b.**
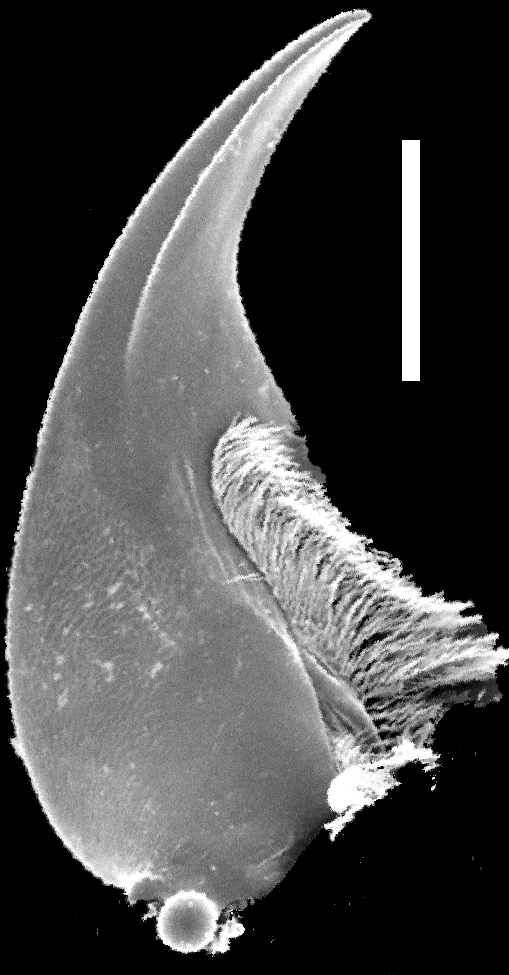
Ventral view of right mandible. Scale bar = 0.25 mm.

**Figure 3c.**
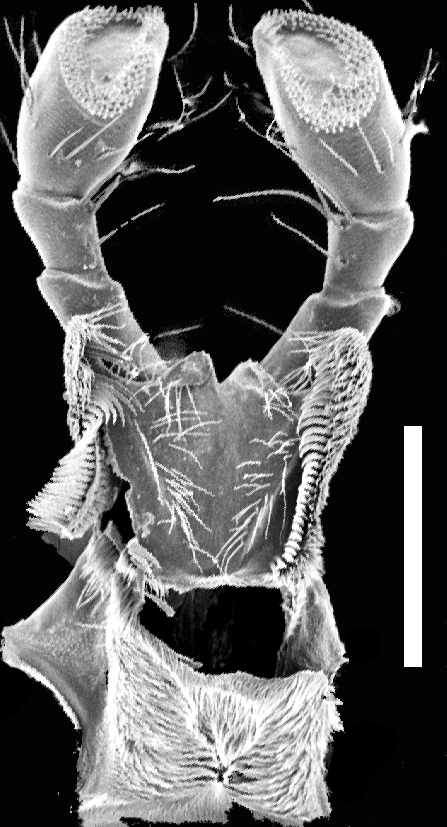
Ventral view of labial palpomeres and hypopharynx. Scale bar = 0.28 mm.

**Figure 3d.**
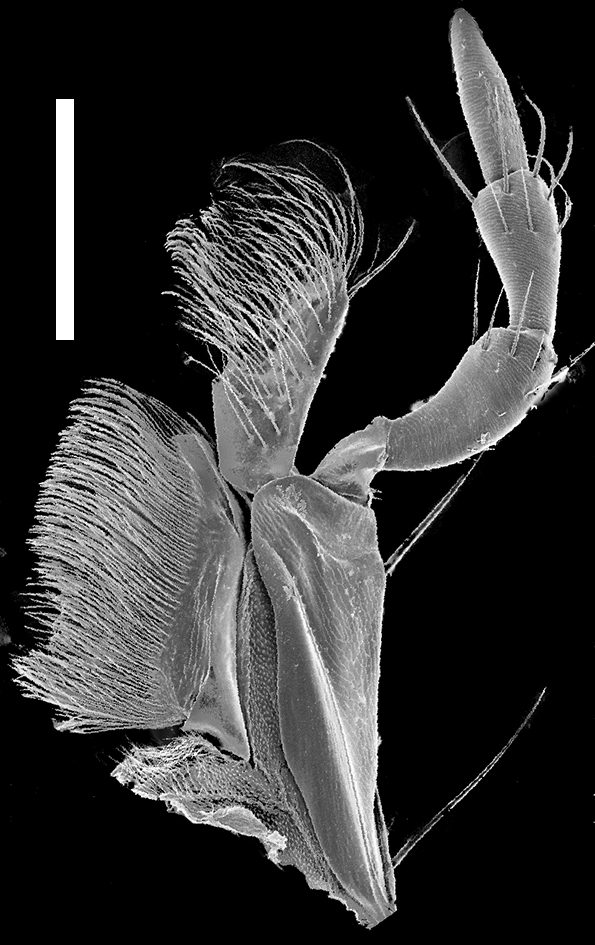
Left maxilla. Scale bar = 0.20 mm.

**Figure 4a.**
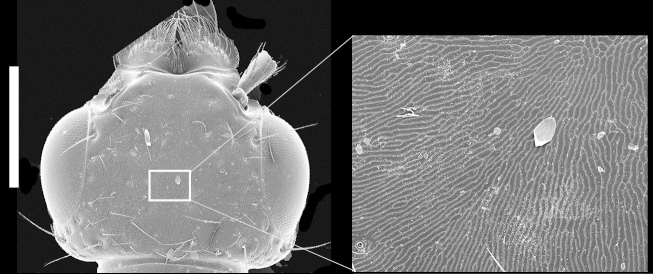
Head, with inset magnification showing the wavy microsculputre at 240x. Scale bar = 0.7 mm.

**Figure 4b.**
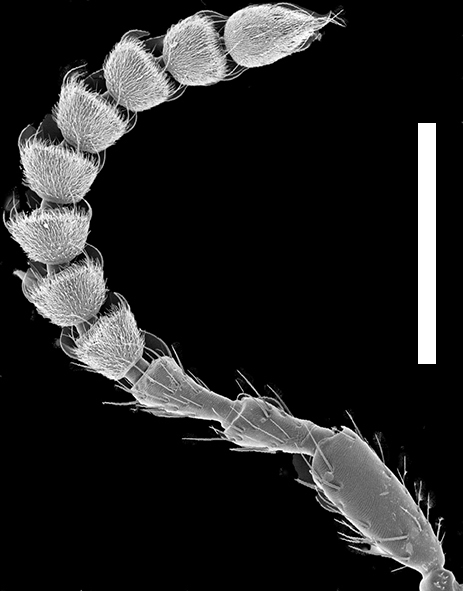
Antenna. Scale bar = 0.7 mm.

**Figure 4c.**
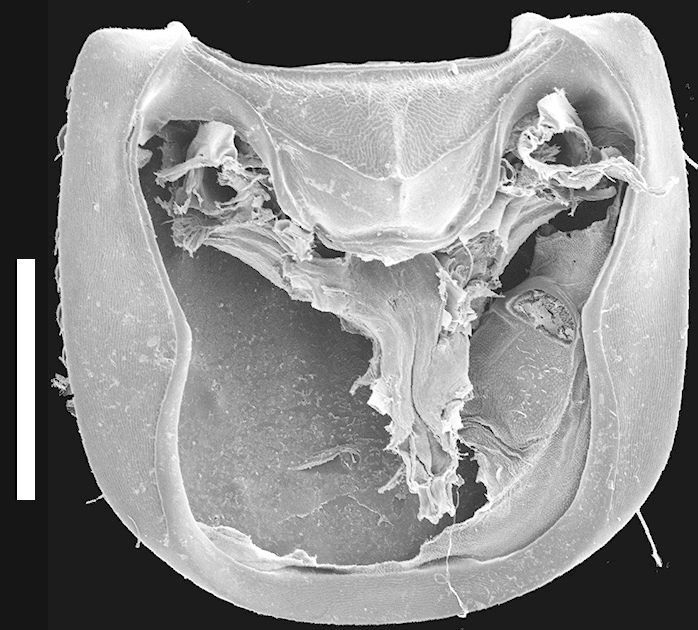
Prosternum and pronotal hypomeron. Scale bar = 0.7 mm.

**Figure 4d.**
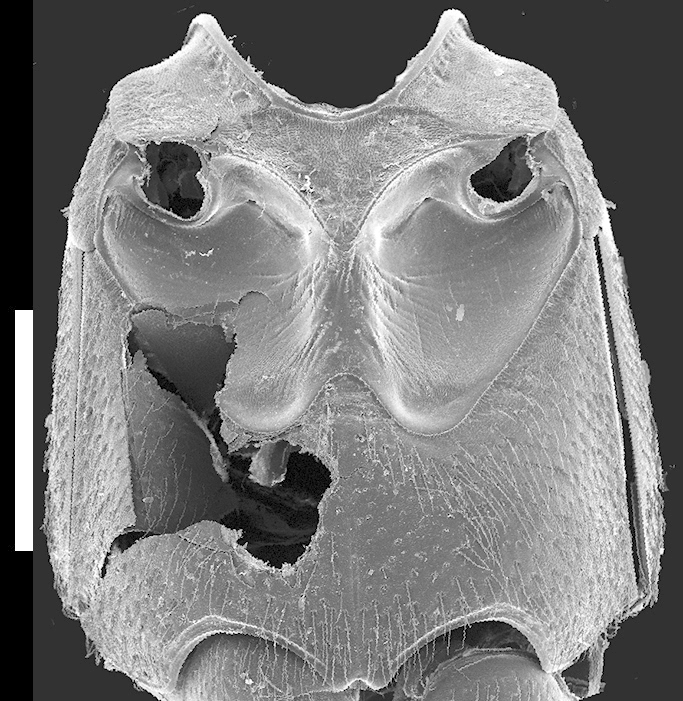
Pterothorax, ventral. Scale bar = 0.9 mm.

**Figure 5a.**
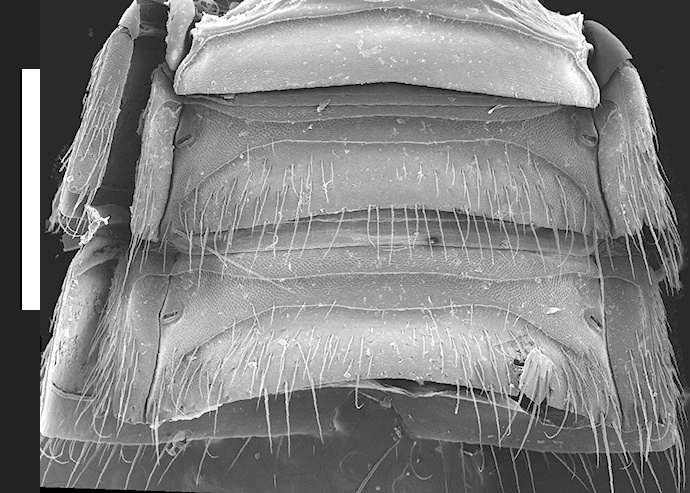
Tergites II-IV. Scale bar = 1.1 mm.

**Figure 5b.**
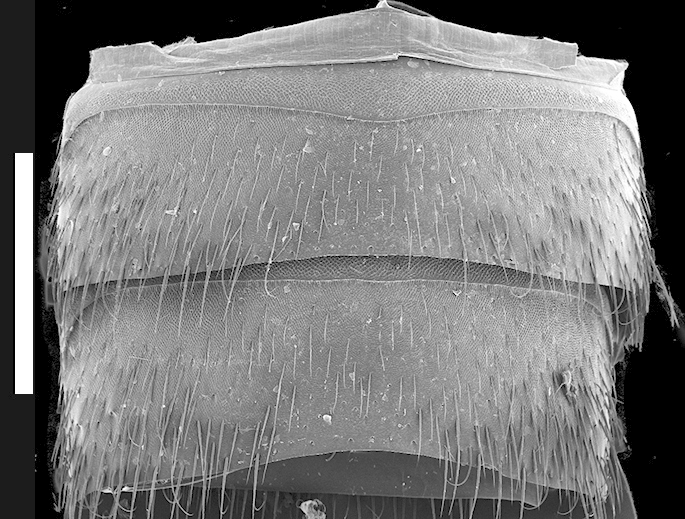
Sternites III-IV. Scale bar = 1.1 mm.

**Figure 5c.**
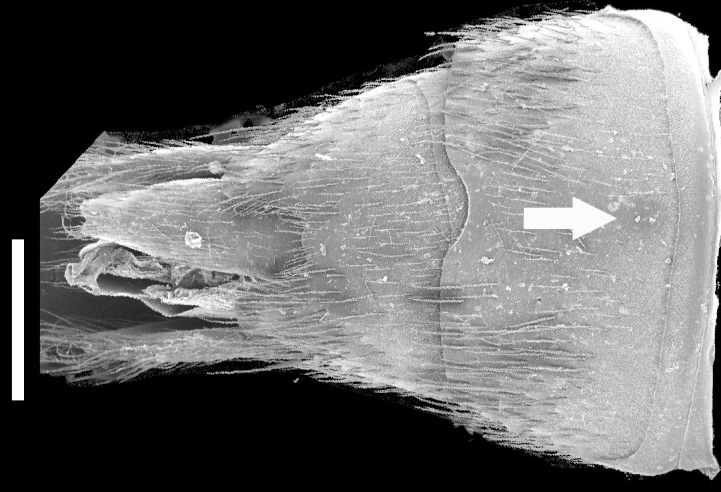
Sternites VII-IX in males. Arrow points the location of the porose structure on VII. Scale bar = 0.7 mm.

**Figure 5d.**
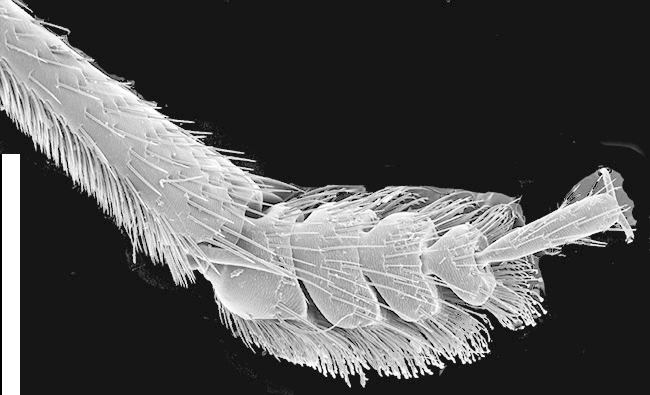
Part of protibia and protarsus. Scale bar = 0.45 mm.

**Figure 5e.**
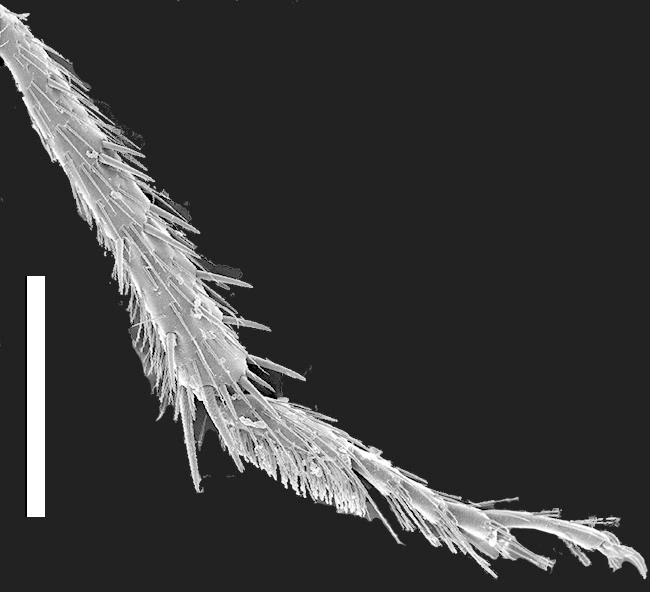
Mesotibia and mesotarsus in males, showing the spatulate setae on tarsomere 1. Scale bar = 0.68 mm.

**Figure 6a.**
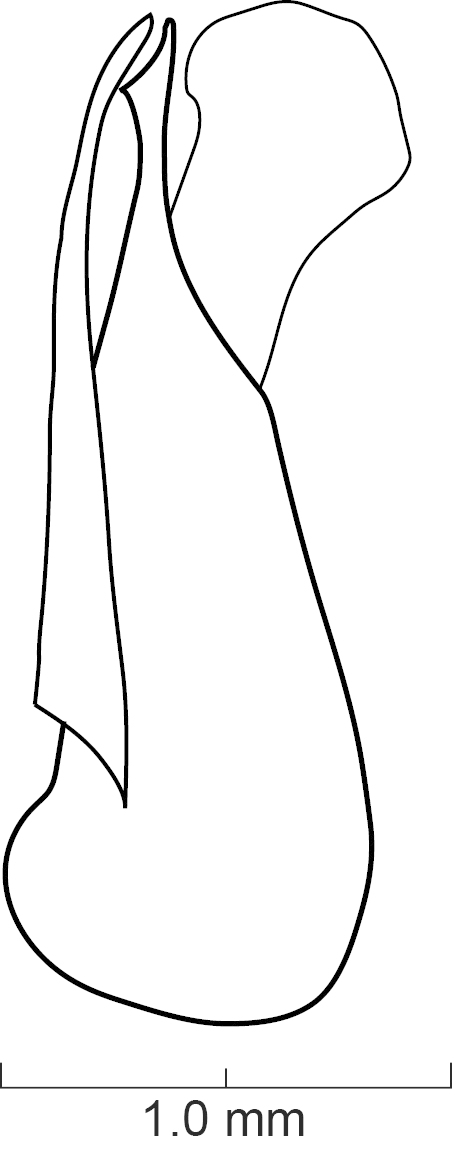
Lateral view.

**Figure 6b.**
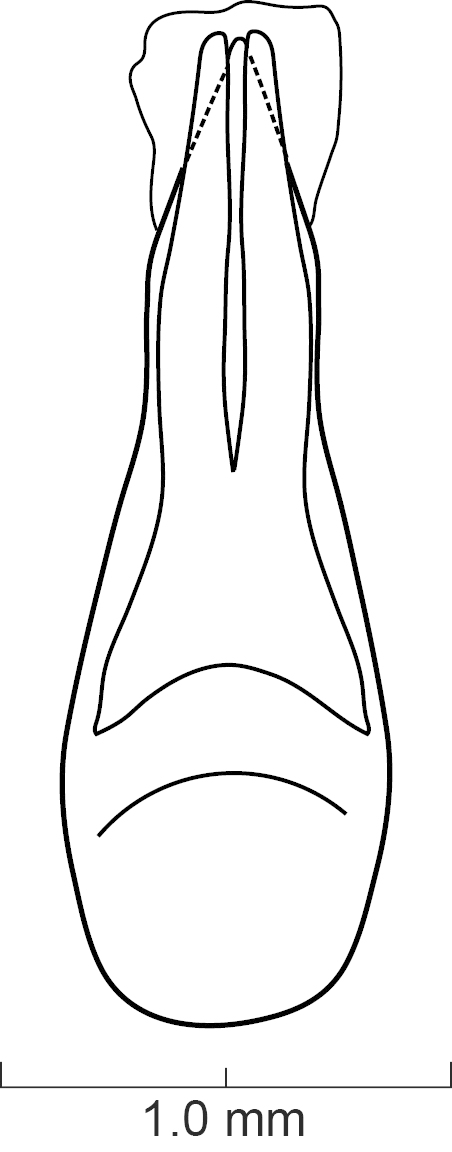
Dorsal view

**Figure 6c.**
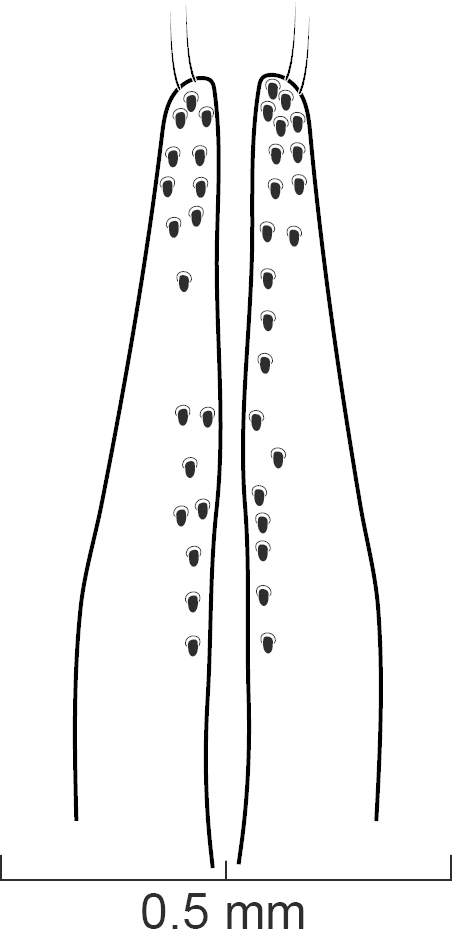
Detail view of the ventral side of parameres.

**Figure 7a.**
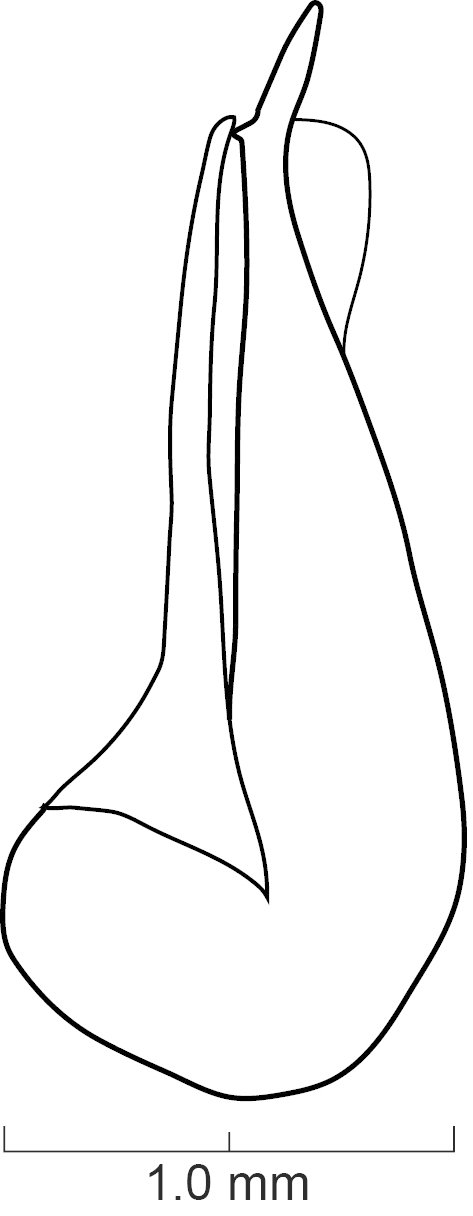
Lateral view.

**Figure 7b.**
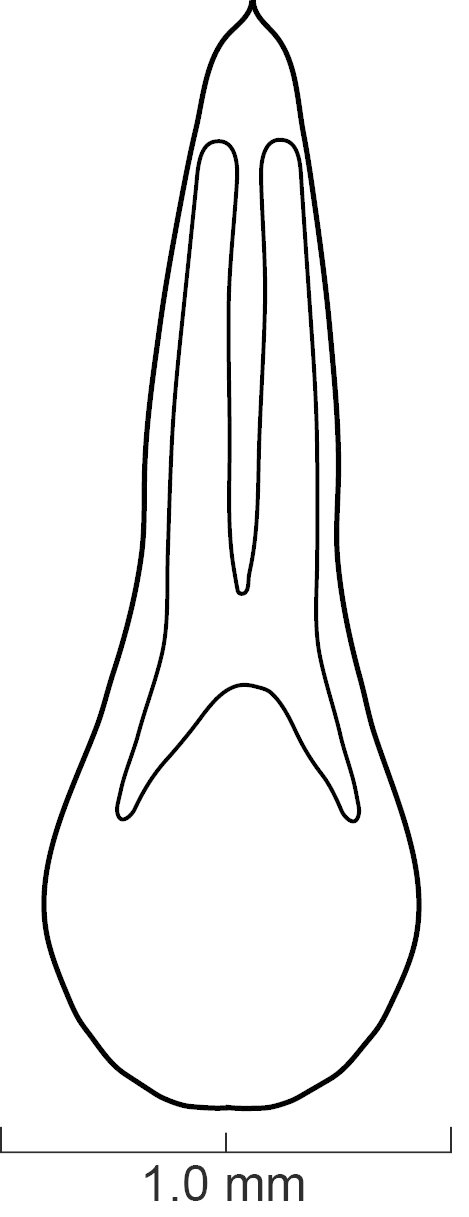
Dorsal view.

**Figure 7c.**
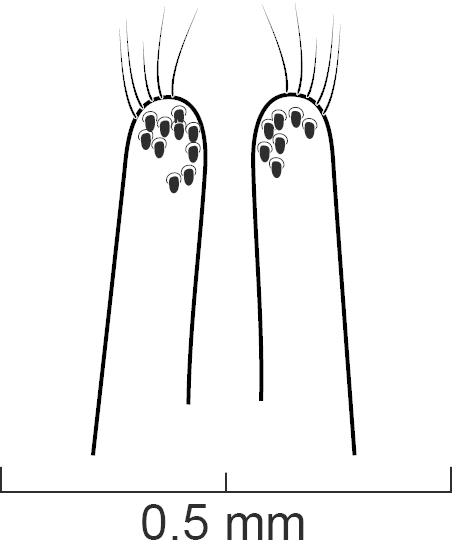
Detailed view of the ventral side of parameres.
